# An advanced 10K SNP panel for genotyping tomato (*Solanum lycopersicum* L.) via targeted genome sequencing

**DOI:** 10.3389/fpls.2025.1582241

**Published:** 2025-05-21

**Authors:** Yawo Mawunyo Nevame Adedze, Yanfen Xu, Song Liu, Yaran Zhao, Changjuan Mo, Renxu Zhang, Jiahui Dong, Haofa Lan, Jingjing Huang, Xingming Chen, Xuefei Gao, Qingzhen Yin, Jianan Zhang

**Affiliations:** ^1^ Reagent Research and Development Center, Molbreeding Biotechnology Co., Ltd., Shijiazhuang, China; ^2^ Institute of Cash Crops, Hebei Academy of Agriculture and Forestry Sciences, Shijiazhuang, China

**Keywords:** optimization, efficiency, genetic, diversity, resistance, tomato

## Abstract

**Introduction:**

Recent breakthroughs in genomics have facilitated the identification of single nucleotide polymorphisms (SNPs) and small insertions-deletions (InDels). With the reduction in sequencing costs, a variety of genotyping tools have emerged for genetic analysis in plants. However, there is a significant need for an effective and affordable tool that combines both foreground and background sites.

**Methods:**

To meet this requirement in tomatoes, four SNP databases accounting for 12,442 SNPs were integrated with 186 trait-specific markers. A total of 335 tomato samples were used for the genotyping by target sequencing analysis. A series of criteria were performed for site selection and for assessing the sequencing data effectiveness.

**Results and discussion:**

The panel designated as the GenoBaits Tomato 10K panel ultimately comprised 11,174 background sites, with 74.83% sourced from database 1 upon optimization. The uniformity_50 and capture efficiency of this panel were 98.03% and 74.84%, respectively, while the SNP detection rate was 99.34%. The SNPs with a minor allele frequency (MAF) > 0.05 accounted for 60.57%, and those with MAF > 0.4 represented 20%. The average genome MAF was 0.18, with a gap value of 0.07 Mbp. The GenoBaits Tomato 10K panel has demonstrated its effectiveness in assessing genetic diversity, with minimal impact from trait-specific markers. This panel effectively pinpointed the predefined resistant and susceptible marker alleles associated with 19 key tomato resistance genes in the targeted population. Therefore, future research should validate them in order to unlock the full diagnostic potential of this panel in tomato genetics and breeding.

## Introduction

The emergence of next-generation sequencing (NGS) has revolutionized genetic research in biology. This advanced sequencing method has allowed the discovery, evaluation, and validation of genetic markers. In tomato, the publication of the first genome sequence in 2012 (The Tomato Genome Consortium, 2012) paved the way for the re-sequencing of several tomato accessions (Park et al., 2014; Fernandez-Pozo et al., 2015; Wang et al., 2019; Liu et al., 2022). As a result, many genomic resources have been developed for tomato research, including extensive transcriptome databases (Kudo et al., 2017), high-density genetic maps (Tanksley et al., 1992; Foolad, 2007; Sim et al., 2012a), and various types of molecular markers (Fernandez-Moreno et al., 2013). Here, three categories of molecular markers can be identified: dominant markers such as Restriction Fragment Length Polymorphism (RFLPs) (Tanksley et al., 1992); co-dominant PCR-based markers, such as simple Sequence Repeats (SSRs) (Bredemeijer et al., 1998; Fernandez-Moreno et al., 2013); and co-dominant high-throughput (HT) markers, like Single Nucleotide Polymorphisms (SNPs) (Hamilton et al., 2012). The SNPs offer the advantage of being utilized on HT genotyping platforms, particularly with the emergence of Next Generation Sequencing (NGS). Genome-wide SNPs have been discovered in various crop species, such as rice (Jimenez-Gomez and Maloof, 2009), maize (Labate et al., 2009), durum wheat (Steemers et al., 2006), sugarcane (Ganal et al., 2011), soybean (Zhao et al., 2011), potato (Thomson et al., 2012), and tomato (Hamilton et al., 2012; Shirasawa et al., 2013; Hirakawa et al., 2013; Bhandari et al., 2021, 2023).

With progress in biological research, various SNPs have been transformed into SNP chips designed for automated high-throughput genotyping platforms, such as fixed and liquid SNP chips. Fixed SNP arrays have been designed for various crops, including rice (Chen et al., 2014), maize (Unterseer et al., 2014), soybean (Song et al., 2013), cotton (Hulse-Kemp et al., 2015), and wheat (Winfield et al., 2016). However, the DNA probes on these arrays are permanently attached, rendering them non-modifiable (Liu et al., 2022). In tomato, the 7720 scorable SNPs have been developed using a transcriptomic SNP database of 62,576 SNPs (Hamilton et al., 2012; Sim et al., 2012a), which have been extensively exploited for genotyping and SNP chips development (Sim et al., 2012b; Rodríguez et al., 2013; Hirakawa et al., 2013; Ruggieri et al., 2014). In contrast, liquid chip is flexible, cost-effective, and require fewer facilities than fixed arrays (Xu et al., 2020). Currently, various liquid-phase chips have been created for numerous crop plants, including GenoBaits Maize 20K (Guo et al., 2019), GenoBaits Rice 10K (Hussain et al., 2022), GenoBaits Soy40K (Liu et al., 2022), GenoBaits Wheat 16K (Huang et al., 2022) and tomato (Sierra-Orozco et al., 2021; Agriplex Genomics, 2023). However, few liquid SNP chips are commercially available for tomato. Despite these advancements, there is limited knowledge regarding the availability of a high-efficiency, cost-effective tomato liquid SNP chip that combines both foreground and background loci for tomato breeding.

Tomato (*Solanum lycopersicum* L) is one of the most important vegetables worldwide. China is the leading producer of tomatoes, generating an impressive 68.34 million tonnes. However, it is noteworthy that the country does not rank among the top 10 nations in terms of yield per hectare (https://ourworldindata.org/; https://faostat.fao.org). This gap can be attributed to various challenges within the Chinese tomato industry, including multiple stress factors and changing consumer preferences. At the same time, there is a shortage of potential elite tomato varieties and effective breeding technologies to develop them. Notably, several important genes linked to pathogen resistance, stress tolerance, and desirable agronomic traits have been successfully identified, as discussed in the literature (Gebhardt, 2023; Wang et al., 2024). Molecular markers associated with those traits have been developed and utilized in breeding programs, including pathogen resistance (Van Ooijen et al., 2007; Lee et al., 2015; Hanson et al., 2016), cold tolerance (Guo et al., 2024; Shah et al., 2024), heat stress tolerance (Sun et al., 2024; Hu et al., 2020), and salinity stress tolerance (Wang et al., 2020, 2021). Further, markers associated with consumer’s preferences, including tomato shelf-life (Yogendra et al., 2013), pigment content (Kang et al., 2017; Yoo et al., 2017; Manoharan et al., 2017), sugar content (Charles et al., 2005), yield (Li et al., 2023), as well as taste and flavor (Rodríguez et al., 2011; Pereira et al., 2021) are readily available online.

These markers have the potential to be a sophisticated genetic resource for the selection of preferred tomato varieties (Yang and Francis, 2005; Majid et al., 2012). When combined with background markers, they can expedite backcross breeding (Carbonell et al., 2018; Kwabena et al., 2022), gene pyramiding and genomic selection (Duangjit et al., 2016; Prabhandakavi et al., 2021; Cappetta et al., 2021). This study intended to create a SNP panel that integrates background markers alongside pathogen resistance, stress tolerance, and consumer preferences in tomatoes. To develop this SNP panel, various tomato SNP databases and gene-based markers associated with those desirable traits have been compiled. Using the GenoBaits system of the GBTS platform of Molbreeding Biotechnology Co., Ltd, the GenoBaits 10K panel has been successfully developed for use in tomato.

## Materials and methods

### Plant materials

In this study, a total of 335 fresh market tomato samples, which included recombinant inbred lines (RIL) and commercial hybrid varieties were utilized. From this collection, 136 tomato samples were used to select the background SNPs, while 199 samples were used to genotype background and foreground sites. Among the 199 samples, 94 samples was specifically select to conduct the comparative analysis of resistance testing. The tomato samples were accessed via a collaborative initiative, including two tomato seed companies and one research institute. All samples were grown in the greenhouses, and 40 day-old-leaves were collected for GBTS analysis.

### Probes design and preliminary marker panel preparation

To develop the tomato 10K panel, four different SNP databases were exploited. Database 1 included 8,744 SNPs from the Tomato SNPs Illumina’s Infinium SNP chip assay, which was created during the Solanaceae Coordinated Agricultural Project (SolCAP) (Sim et al., 2012a). Database 2 was composed of 680 SNPs identified by Hirakawa et al., 2013). Database 3 accounting for 1,248 SNPs was derived from GWAS analysis (Shirasawa et al., 2013). Database 4 accounting for 1,552 SNPs was identified from whole genome re-sequencing data accessible on NCBI (SRP150040). A total of 12,224 SNP sites were compiled from those databases as background SNPs while 218 sites associated with disease resistance, abiotic stress tolerance, fruit quality and other beneficial traits were grouped as traits-specific markers (TSM). DNA probes were developed using the Heinz1706 genome sequence (version SL2.40) as a reference. The design criteria included a homology number< 3 for background sequences and< 5 for trait-specific loci for each DNA probe. The GC content was set between 30% and 70% as described by Guo et al., 2021. To improve capture efficiency, a 110bp double-stranded DNA probes was designed (Zhou et al., 2021). Probes were synthesized and mixed using the GenoBaits system of Molbreeding Biotechnology Co., Ltd. in Shijiazhuang, China.

### GBTS analysis and background SNP selection

Genomic DNA was extracted from tomato leaves using the Plant Genomic DNA Extraction Kit of Molbreeding Biotechnology Co., Ltd (GenoPrep v2.0). The purity and integrity of the extracted DNA were assessed through 1% agarose gel electrophoresis, while the DNA concentration was accurately measured using a Qubit. Genomic DNA (≥200 ng per sample) was fragmented using an ultrasonic Crusher (Ultrasonic Crusher Q800R3, Qsonica Co Ltd, USA) to achieve average DNA fragment sizes ranging from 200 to 500 base pairs (bp). DNA library was prepared using the DNA Library Prep Kit for ILM (GenoBaits v4.0). Fragmented DNA underwent a series of processing such as DNA end repair, adenylation, adapter ligation using GenoBaits End Repair Enzyme, GenoBaits Ultra DNA ligase and GenoBaits Adapters for ILM. The ligated DNA fragments were further connected to the appropriate Barcodes for ILM and amplified using GenoBaits^®^ PCR Master Mix. The products were finally purified using GenoPrep^®^Clean Beads for Genomic DNA following the instruction manuals of Molbreeding Biotechnology Co., Ltd. The paired-end DNA library was then captured with the 10K tomato marker panel at 65°Cusing the DNA Hybridization Kit for ILM (GenoBaits v3.3). The paired-end DNA library was enriched using GenoBaits DNA probe beads and sequenced on an Illumina Hiseq X Ten PE150 sequencer with a sequencing depth of 100-fold. The raw data underwent quality filtering using FASTQ software (Chen et al., 2018) and were subsequently aligned to the Heinz1706 reference genome with BWA (version 0.7.10-r789 software (Li et al., 2009). SNP calling was implemented using the standard pipeline of GATK (version 3.1) software (Poplin et al., 2018). The criteria for background SNP selection were: nucleotide missing rate of< 0.1, uniformity> 0.25, a target rate>0.5, and those criteria must be satisfied at a depth of 40X for the simulated data. This filtering is imposed to enhance panel capture efficiency.

### Description of GenoBaits working system

GenoBaits relies on the effective capture of targets through a complementary pairing of the probe and the target sequence. Initially, the double-stranded DNA probes were biotinylated through the attachment of biotin protein. Next, the probe was hybridized with the target sequences, resulting in the formation of double-stranded DNA from the constructed genomic DNA libraries. Further, streptavidin-coated magnetic beads were employed to capture the biotin-labeled probe, thereby isolating the target sequence. Finally, the captured sequences were subjected to elution, amplification, and sequencing. Uniformity is assessed by the percentage of captured regions (number of reads) that reach 10%, 20%, and 50% of the average depth across the loci in relation to all regions. Capture efficiency indicates the proportion of useful data (targeted regions) relative to the total sequencing data. To improve both capture efficiency and uniformity, the hybridization reagents and wash buffer were optimized. Further, the GBTS cost was evaluated per sample, including the required next-generation sequencing (NGS) volume, the cost for DNA extraction, library preparation and hybridization capture analysis.

### Panel optimization procedure

**Figure 1:**
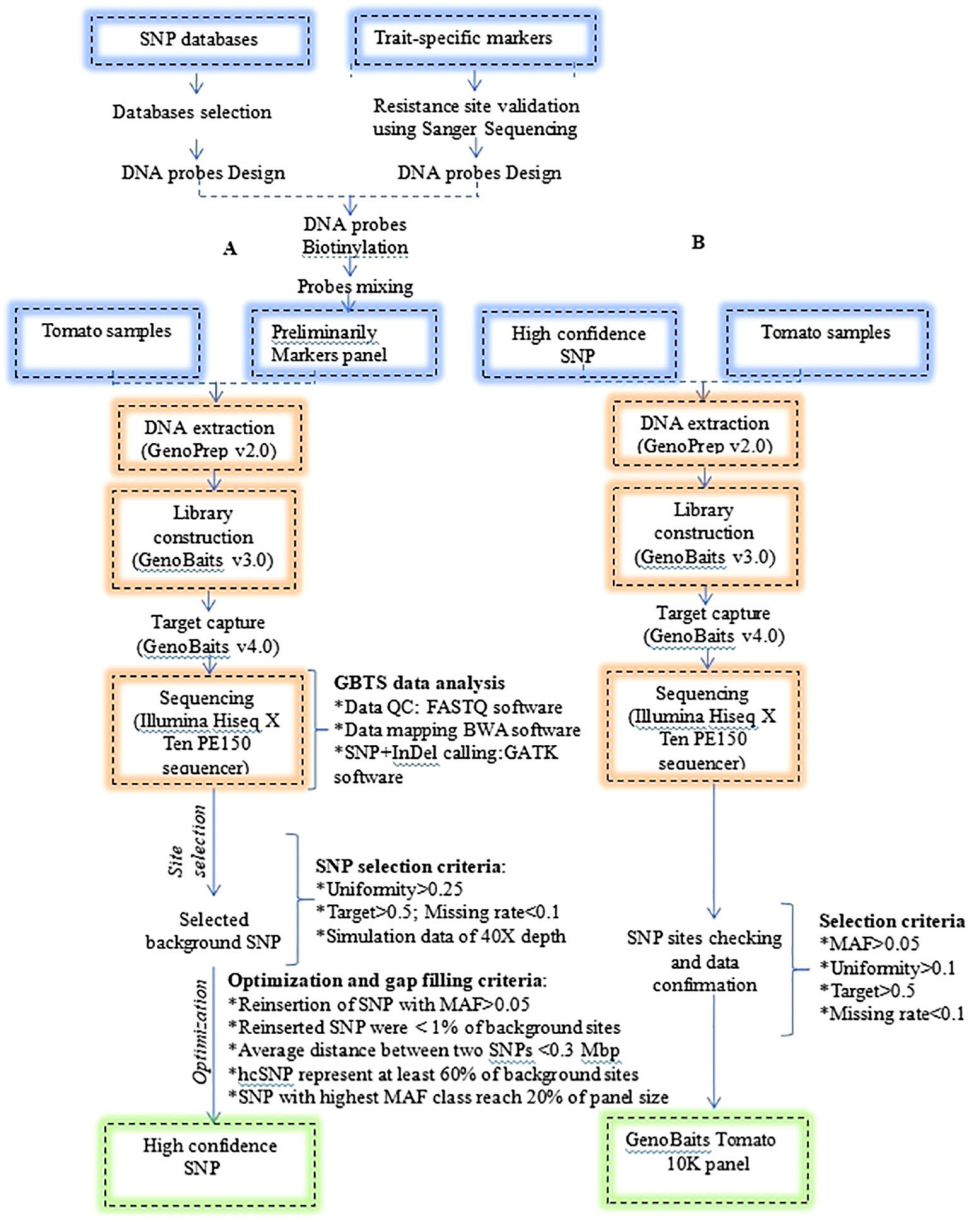
Development procedure of the GenoBaits Tomato 10K panel. **(A)** indicates the process of developing and optimizing the background SNP to identify high confidence SNP. **(B)** indicates the procedure for confirming the optimized SNP sites. Please note that the selection criteria were not applied to the trait-specific marker sites (SNP and InDel); this procedure pertains solely to the background SNP sites.

Criteria for optimization were as follows: (1) consideration of SNPs with MAF > 0.05 from the discarded SNP dataset during the first selection, (2) the maximum average distance between adjacent SNPs on each chromosome should not surpass 0.3 Mbp, (3) the total number of the reinserted SNPs should account for less than 1% of the background SNPs, (4) SNPs with MAF > 0.05 should account for at least 60% of the background SNPs, and (5) SNPs with the highest MAF threshold class should reach 20% of the total panel size. The development and optimization procedure of the GenoBaits Tomato 10K panel was represented in **Figure 1**.

### Genetic diversity analysis

To assessed genetic diversity (GD), several metrics were considered: observed allele number (Ao), effective allele number (Ae), observed heterozygosity (Ho), expected heterozygosity (He), and polymorphism information content (PIC). The PIC values were categorized and interpreted as described by Serrote et al. (2019): low PIC (0-0.10), medium PIC (0.10-0.25), high PIC (0.3-0.4), and very high PIC (0.4-0.5). The following formula was employed to calculate allele frequency and PIC (Equation 1):


(1) $$PIC=1-\sum_{i=1}^{n}P_{i}^{2}-\sum_{i=1}^{n-1}\sum_{j=i+1}^{n}2P_{i}^{2}P_{j}^{2}$$

which: pi and pj are allele frequencies at alleles i and j, and n is the number of alleles, respectively.

### Phylogenetic and population structure analyses

To establish connection between the 335 samples, a phylogenetic tree was constructed using the neighbor-joining method with the Kimura 2-parameter/p-distance model in MEGA-X software (www.megasoftware.net), incorporating 1000 bootstrap replicates and the resulting tree was visualized using MEGA-X. Principal component analysis (PCA) was conducted using GCTA (v1.92.4) software, as described by Lu et al. (2024). We calculated the variance explained by each principal component (PC) and created a score matrix for each sample across the PCs. Background SNPs and trait-specific markers were separately used to conduct a population structure analysis using ADMIXTURE (v1.22). The determination of the number of sub-populations was performed through K-fold cross-validation, with various K values reflecting the estimated number of sub-populations. Stacked assignment bar plots of the Q matrix for each K value were generated using the R package Pophelper (http://royfrancis.github.io/pophelper). The optimal number of clusters was determined by examining the cross-validation error (CV error), with the K value yielding the lowest CV error indicating the most appropriate number of clusters.

### Kinship and linkage disequilibrium analyses

Kinship refers to the genetic relatedness between accessions, as well as the relative genetic relatedness among any accessions. The GCTA software (version 1.92.1: Yang et al., 2011) was utilized to estimate kinship among the tomato samples. The mean expected variance of SNPs was employed to adjust the expected variance, resulting in a heatmap of the kinship G matrix. The genetic relatedness of the samples was assessed based on the limited interval values established by Weir et al. (2006) and Kristen et al. (2011). Meanwhile, the linkage disequilibrium analysis was performed for all possible pairs of high-confidence SNPs, examining genome-wide loci separately from those linked to trait-specific loci. LD decay between markers was quantified using the parameter r^2^ (Hill and Robertson, 1968) estimated using Haploview software (version 4.2: Barrett et al., 2005). The pairwise r^2^ values were calculated for all SNPs in a 500-kb window. Then, average LD was calculated in increments of 1 kb according to marker distances. Finally, LD decay distances were profiled using the ggplot2 package in the R language.

### Comparison of the molecular resistance screening analyses

To confirm the informativeness of the trait-specific marker sites, the genotyping results obtained in this work were compared with results from two external laboratories, using 94 meticulously chosen samples believed to harbor the resistance genes. A detection rate of 90% was established to confirm the presence of these genes (Data not shown).

## Results

### High confidence tomato SNPs accessibility

**Figure 2:**
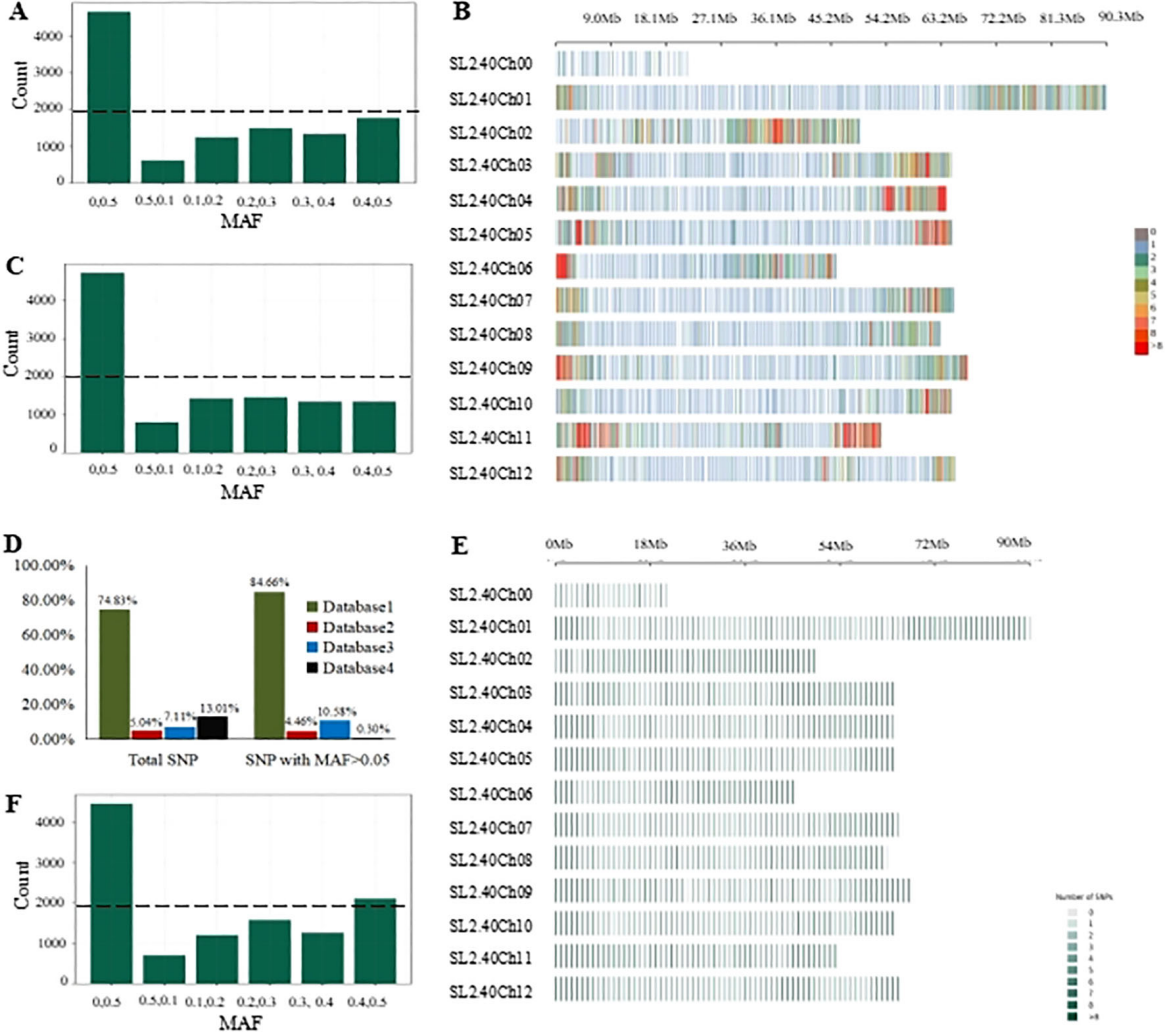
Genome-wide characterization of SNP in the GenoBaits Tomato 10K panel. **(A)** Categorization of candidate SNP data following different MAF threshold levels; **(B)** Chromosome distribution of the candidate SNP datasets; **(C)** Categorization of high capture-efficiency SNP data following different MAF threshold levels. **(D)** A graphical illustration of the contribution of each SNP database to the candidate SNP datasets and SNP data with MAF > 0.05; **(E)** Chromosome distribution of high-confidence SNP data. **(F)** Categorization of high-confidence SNP based on different MAF threshold levels. A dotted line indicates the number of SNP with the highest MAF values (0.4, 0.5) within the three SNP datasets.

Comprehensively, 12,442 marker sites were obtained, including the background SNPs and trait-specific markers sites. Probes were successfully designed for 11,473 sites. In average, 2 probes per site for the background SNPs and 3 probes per site for the trait-specific sites. All these probes were mixed to generate a preliminary tomato 10K panel. To examine the performance of this panel, GBTS analysis was conducted using 136 tomato varieties. An average clean data of 887.09 Mbp and mapped data of 825.04 Mbp were obtained while the uniformity, 20, 50 and capture efficiency were 97.02%, 78.03% and 68.99% respectively (**Table 1**; **Supplementary Table 1**). Further, SNPs were categorized based on the various MAF threshold classes. SNPs with a MAF value > 0.05 accounted for less than 60% of the background SNPs while those with a MAF value >0.4 were less than 20% of the total panel size (**Figure 2A**). However, they achieved high-density genome coverage with an average MAF of 0.17 ± 0.17 (**Figure 2B**). To improve the panel, SNP selection was performed, resulting in the retention of 11,096 sites, which included 10,910 background SNPs according to the defined selection criteria. These were designated as high confidence SNPs (hcSNPs). Among them, 6,448 SNPs exhibiting a MAF value > 0.05 were primarily sourced from SNP database1. Unfortunately, SNPs with a MAF value > 0.05 were less than 60% of the background SNPs, and those with a MAF accounted for less than 20% of the overall panel size (**Figure 2C**). Meanwhile, trait-specific marker sites with missing data were discarded, leaving a total of 186 sites. Virtually, the selected SNPs were experimentally genotyped using a group of 199 tomato varieties (**Supplementary Table 2**). The average SNP detection rate across the two distinct experiments was 99.34% (**Supplementary Table 2**). The number of SNPs with a MAF value > 0.05 was 6,376, accounting for 58.40% of the background SNPs. Notably, the uniformity rose to 98.03%, and the on-target value increased to 74.84%, indicating a significant improvement of the panel (**Table 1**; **Supplementary Table 2**) with a genome wide average MAF of 0.18 ± 0.18.

**Table 1:** Average sequencing data performance of 335 tomato varieties.

Sample size	Clean data (Mbp)	Mapped data (Mbp)	Uniformity_10 (%)	Uniformity_20 (%)	Uniformity_50 (%)	On_target rate (%)	Effective data rate (%)	Percentage duplication (%)
136	887.09	825.04	99.08%	97.02%	78.03%	67.99%	63.32%	6.76%
Sample size	SNP Detection rate (%)	InDel Detection rate (%)	Overall Detection rate (%)	Uniformity_10 (%)	Uniformity_20 (%)	Uniformity_50 (%)	On_target rate (%)	Percentage duplication (%)
199	99.37%	85.41%	99.34%	99.26%	98.03%	76.61%	74.84%	1.79%

Mbp indicates Megabasepair.

### Optimization and current status of the 10K tomato panel

The optimization aimed to enhance the number of SNPs with MAF > 0.05 while ensuring a consistent distribution across the chromosomes. Here, new criteria were imposed on previously discarded SNPs. Consequently, 392 SNPs with a MAF > 0.05 were reintroduced to the panel, bringing the total number of sites to 11,360. This total comprises 11,174 background SNPs, of which 6,768 are polymorphic, along with 186 trait-specific markers (**Table 2**). The average distance between adjacent markers across the 12 tomato chromosomes varied from 0.04 Mbp on chromosome 1 to 0.25 Mbp on chromosome with a mean value of 0.07 (**Table 3**). The optimized panel was referred to as the GenoBaits Tomato 10K panel. In terms of the overall background SNPs, 74.83% originated from database 1 while for SNPs with a MAF > 0.05, 84.66% came from the same database (**Figure 2D**). Within the databases used, database 3 had the relatively higher percentage of SNPs with MAF > 0.05, reaching 90.06%. This was followed by database 1, database 2, and database 4, which scored 68.52%, 53.64%, and 41.38% as the percentage of SNPs with MAF > 0.05, respectively (**Table 3**). The tomato 10K panel demonstrated impressive performance metrics and exhibited a well-balanced distribution across chromosomes. (**Figure 2E**). Comprehensively, it displayed 60.57% of SNP with MAF > 0.05 while the percentage of SNP with MAF >0.4 was 20% (**Figure 2F**). The estimated cost for the GBTS included 1.5 RMB (0.21 USD) for DNA extraction, 3.5 RMB (0.48 USD) for library construction, 40 RMB (5.5 USD) for hybridization capture, and 5.4 RMB (0.74 USD) for NGS, with a total NGS volume of 0.54G. This brought the overall cost to 50.4 RMB (6.93 USD) per sample.

**Table 2:** Summary of genetic markers in each database and proportion of SNP with MAF>0.05.

SNP data sources	All marker sites	MAF≦0.05	SNP with MAF>0.05	Relative proportion of SNP with MAF>0.05
Database 1	8362	2632	5730	68.52%
Database 2	563	261	302	53.64%
Database 3	795	79	716	90.06%
Database 4	1454	1434	20	1.38%
Total	11174	4406	6768	60.57%

MAF indicates minor allele frequency.

**Table 3:** Average mean value of chromosome wide gap distance.

Chromosome	Length (Mbp)	Min_gap (bp)	Max_gap (Mbp)	Mean_gap (Mbp)
SL2.40ch01	90.30	1	0.75	0.10
SL2.40ch02	49.92	1	0.86	0.04
SL2.40ch03	64.84	1	0.73	0.07
SL2.40ch04	64.06	1	0.83	0.05
SL2.40ch05	65.02	1	0.75	0.06
SL2.40ch06	46.04	1	0.67	0.05
SL2.40ch07	65.27	1	0.65	0.09
SL2.40ch08	63.03	1	0.60	0.10
SL2.40ch09	67.66	0	1.10	0.08
SL2.40ch10	64.83	9	0.73	0.10
SL2.40ch11	53.39	0	0.55	0.04
SL2.40ch12	65.49	1	0.69	0.10
Average	63.32	1.50	0.74	0.07

Mbp indicates megabasepair.

### Genetic diversity assessment using the genobaits tomato 10K panel

To determine the potential of the panel for exploring genetic diversity in tomatoes, six parameters were evaluated such as MAF, PIC, Ao, Ae, Ho, and He using the 335 samples. The parameters were estimated using both the 10K panel and the trait-specific markers separately. As a result, the 10K panel displayed average values and standard deviations of 0.16 ± 0.17, 0.17 ± 0.15, 1.75 ± 0.44, 1.37 ± 0.38, 0.05 ± 0.06, and 0.21 ± 0.20 while the trait-specific markers scored the values of 0.15 ± 0.16, 0.17 ± 0.15, 1.74 ± 0.51, 1.35 ± 0.37, 0.06 ± 0.08, and 0.21 ± 0.20 for MAF, PIC, Ao, Ae, Ho, and He, respectively (**Table 4**).It was observed that the PIC and He values were identical for both marker types (**Table 4**), indicating a consistency in results between the trait-specific markers and the 10K panel in the analysis of genetic diversity.

**Table 4:** Average and standard deviation values of genetic diversity indicators.

Marker type	Sample size		MAF	PIC	Ao	Ae	Ho	He
High confidence markers	110	Average	0.16	0.17	1.75	1.37	0.05	0.21
		SD	0.17	0.15	0.44	0.38	0.06	0.20
Trait-specific markers	200	Average	0.15	0.17	1.74	1.35	0.06	0.21
		SD	0.16	0.15	0.51	0.37	0.08	0.20

SD indicates standard deviation, MAF minor allele frequency, PIC polymorphism information content, Ao observed allele, Ae expected allele, Ho observed heterozygosity, and He indicates expected heterozygosity.

### Phylogenetic tree and population structure assessment using the genobaits tomato 10K panel

**Figure 3:**
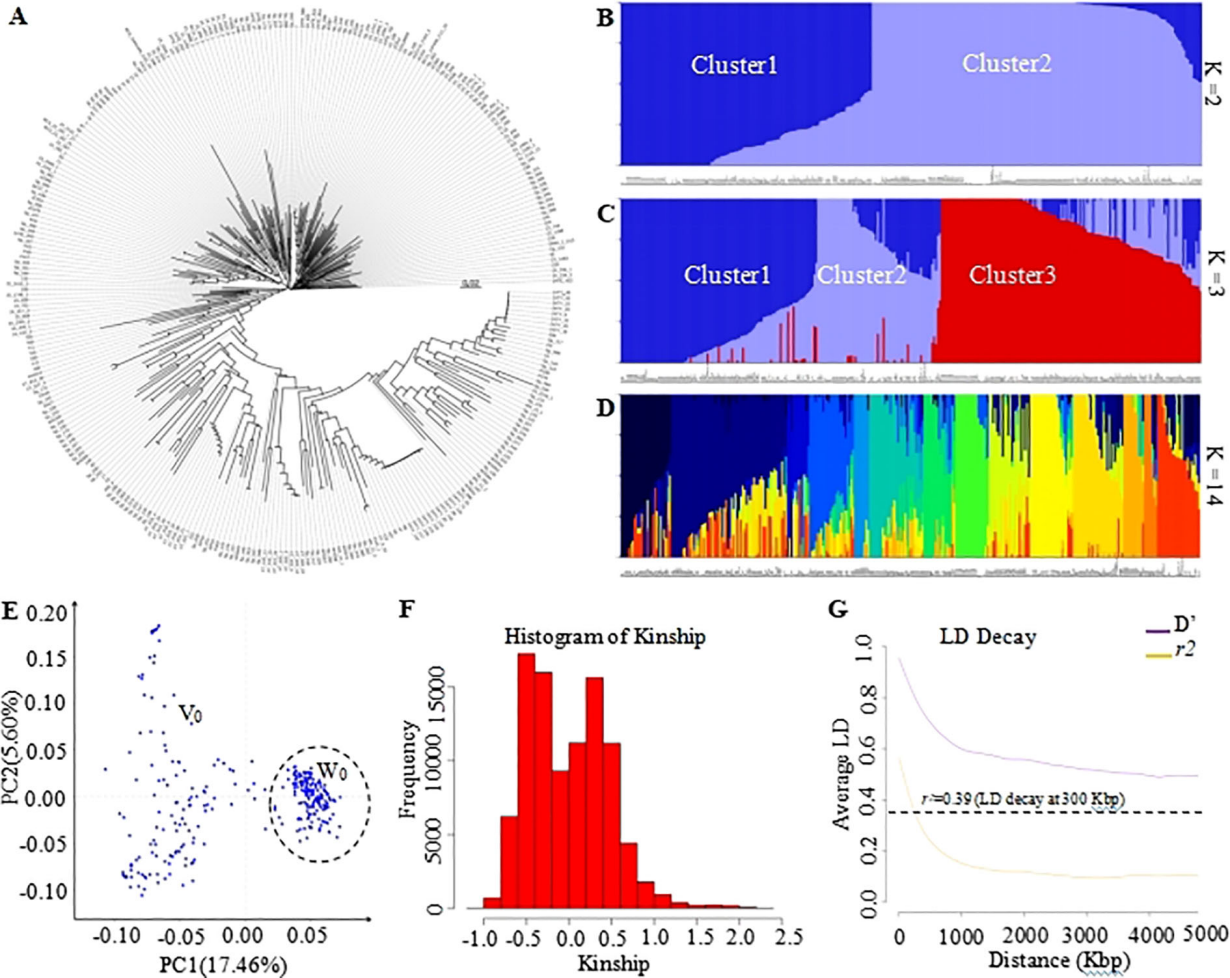
Display of genetic variation of tomato samples using the GenoBaits Tomato 10K panel.**(A)** Subgroups obtained using the phylogenetic analysis, population structure analysis **(B–E)** principal component analysis. Presentation of population structure results with K = 2 **(B)**, 3 **(C)**, and 14 **(D)**, as examples. Graphical representation of Kinship results **(F)** and Linkage Disequilibrium analysis **(G)**. PC1 denotes the first principal component, which accounts for the highest variance, while PC2 represents the second principal component, indicating the second highest variance explained. The intensity of linkage disequilibrium is represented as D’ or r², with r² indicating the half decay distance. The calculated LD decay threshold value of 0.39, herein corresponds to the average value of the r² values. Cluster1, Cluster2 to Cluster3 are clusters number for K value=2 and 3. V0 and W0 were two tendency subgroups upon PCA analysis.

**Figure 4:**
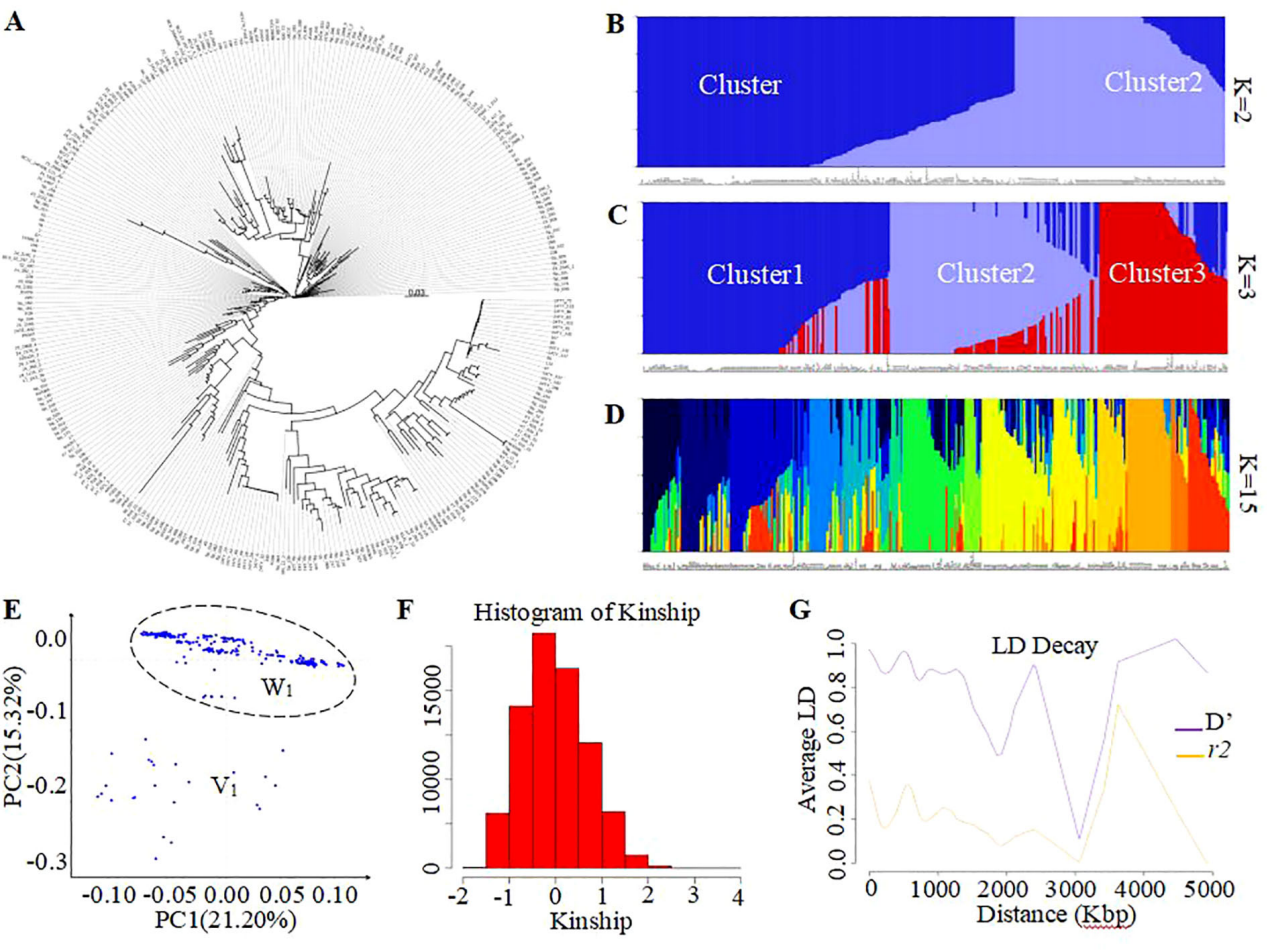
Display of genetic variation of tomato samples using the trait-specific markers. **(A)** Subgroups obtained using the phylogenetic analysis, population structure analysis **(B–D)**, and principal component analysis **(E)**. Presentation of population structure results with K = 2 **(B)**, 3 **(C)**, and 14 **(D)**, as examples. Graphical representation of Kinship results **(F)** and Linkage Disequilibrium analysis **(G)**. PC1 denotes the first principal component, which accounts for the highest variance, while PC2 represents the second principal component, indicating the second highest variance explained. The intensity of linkage disequilibrium is represented as D’ or r², with r² indicating the half decay distance. The calculated LD decay threshold value of 0.39, herein corresponds to the average value of the r² values. C1, C2 to C3 are clusters number for K value=2 and 3. V1 and W2 were two tendency subgroups upon PCA analysis.

To investigate the feasibility of this panel to determine phylogenetic and population structure, the corresponding analyses were performed. As result, the phylogenetic analysis has empirically separated all 335 tomato samples into three GenoBaits Tomato 10K panel and two distinct subgroups using the trait-specific markers, respectively (**Figures 3A**, **4A**). Conversely, structural analysis has produced K values ranging from 1 to 15, regardless of the types of markers employed (see **Supplementary Figures 1A, B**). This suggests that both marker types were capable of categorizing all samples into as many as 15 distinct subgroups. Meanwhile, a comparison was made between the results of the phylogenetic tree and the population structure, with particular emphasis on the K values of 2 and 3 from the structure analysis in spite of the optimal K values of 14 and 15 obtained for the whole SNP panel and trait-specific markers, respectively (**Figures 3B, C**, **4B, C**). the grouping results of tomato samples using the GenoBaits Tomato 10K panel appeared to be consistent with those derived from the trait-specific markers (see **Figures 3A–E**, **4A–E**). Taken together, the GenoBaits Tomato 10K panel proved to be effective for performing phylogenetic and population structure analyses in tomatoes.

### Principal component analysis, kinship, and linkage disequilibrium

To enhance our understanding of the GenoBaits Tomato 10K panel in population study, three separate analyses, such as principal component analysis, linkage disequilibrium analysis, and kinship analysis were conducted. In the PCA results, variance explained (VE) values ranged from 17.46% to 21.20% for PC1 and from 5.60% to 15.32% for PC2 using the GenoBaits Tomato 10K panel and trait-specific panel, respectively. Further, the tendency of two subgroups was shaped using both marker types, which suggests the conformity between their PCA results (**Figures 3E**, **4E**). Nevertheless, the genetic relationships between the samples were not completely clarified with PCA analysis. To elucidate genetic relatedness between samples used, a kinship analysis was done. The kinship values varied from -1 to 2.5 using the GenoBaits Tomato 10K panel and from -2 to 4 using the trait-specific markers (**Figures 3F**, **4F**; **Supplementary Figures 1C, D**). The overlap Kinship values confirmed the strong similarity in the performance of both types of markers. Another key genetic analysis is the linkage disequilibrium (LD). Here, the obtained average r²value of 0.39 indicates that the LD decayed roughly at 300 Kbp for the commercial panel, while for the trait -specific markers the result was abnormal (**Figures 3G**, **4G**). The estimated LD decay distance aligned with the gap threshold value of 0.3 Mbp imposed during the gap-filling process.

### Detection of the trait-specific markers in the genobaits tomato 10K panel

**Figure 5:**
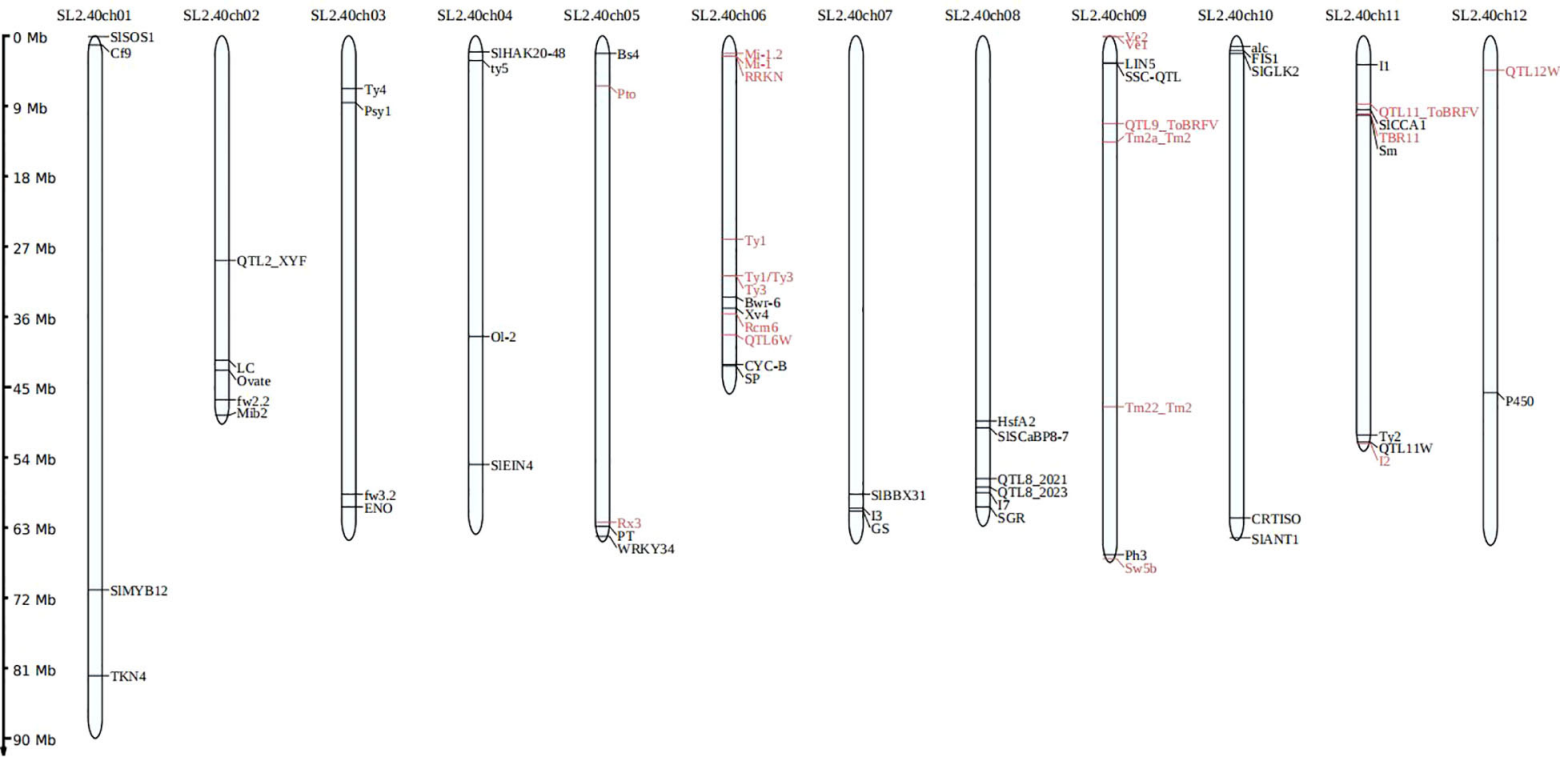
An illustration depicting the locations of trait-specific marker sites across the 12 chromosomes of tomato.

To detect the trait-specific marker sites in the panel, we focused on 199 varieties known to be involved in resistance screening programs. A total of 139 loci were analyzed, including 86 associated with disease resistance, 9 related to abiotic stress tolerance, and 44 linked to fruit quality and agronomic traits, with particular emphasis on the results from the disease resistance markers. In this study, we examined 16 significant diseases associated with 34 resistance genes, averaging around 2 genes per disease (see **Supplementary Table S5**). In this study, we successfully identified 36 resistance gene loci associated with 16 tomato diseases (see **Table 5**; **Supplementary Table S6**), and their locations on the 12 tomato chromosomes are illustrated (**Figure 5**). This panel successfully identified the previously reported resistant and susceptible marker alleles linked to 19 key tomato resistance genes within the integrated trait-specific marker sites, highlighting its exceptional specificity.

**Table 5:** List of resistance genes validated using GenoBaits Tomato 10K panel in this study.

Disease name	Resistance genes	Sites number	Result determination criteria
Tomato brown rugose fruit virus disease	*QTL6W*	3	Haplotype form
	*QTL12W*	4	Haplotype form
	*QTL9_ToBRFV*	4	Haplotype form
	*QTL11_ToBRFV*	4	Haplotype form
	*TBR11*	5	Haplotype form
Tomato yellow leaf curl disease	*Ty1*	5	Haplotype form
	*Ty3*	6	Haplotype form
	*Ty1&Ty3*	3	Haplotype form
Root-knot nematode disease	*RRKN*	3	Haplotype form
	*Mi-1*	1	Single point form
	*Mi-1.2*	1	Single point form
Tobacco mosaic leaf disease	*Tm2a*	2	Haplotype form
Tomato mosaic leaf disease	*Tm22*	3	Haplotype form
Fusarium wilt disease	*I2*	1	Single point form
Verticillium wilt disease	*Ve1*	1	Haplotype form
	*Ve2*	1	Haplotype form
Bacterial canker disease	*Rcm6*	1	Single point form
Bacterial speck disease	*Pto*	1	Single point form
	*Rex3*	1	Single point form
Tomato spotted wilt disease	*Sw5b*	5	Haplotype form

## Discussion

High-throughput genotyping technologies are becoming more prevalent in genetic research and breeding. In this study, we have exploited the existing SNP databases and trait-specific markers to develop the GenoBaits Tomato 10K, which includes 139 loci associated with disease resistance, abiotic stress tolerance, fruit quality, and agronomic traits. This was accomplished using the GBTS technology platform from Molbreeding Biotechnology Co., Ltd. The effectiveness of this panel is demonstrated through genetic diversity analysis conducted on 335 tomato samples. The aim is to promote a high-efficiency tomato SNP panel with accurate detection and reduced sequencing costs. The newly developed panel was named GenoBaits Tomato 10K panel. Previous research has shown that the substitution of RNA probes with DNA probes enhances uniformity, capture efficiency, and overall experimental stability (Guo et al., 2021). Moreover, the probes with a Guanine-Cytidine (GC) content ranging from 30% to 70% have exhibited superior capture capabilities compared to those with a GC content below 30% while the captured genomic regions with a GC content exceeding 70% have adversely affected PCR performance during targeted sequencing. Here, we have selected double-stranded DNA probes with a GC content ranging from 30% to 70% for panel preparation. As anticipated, the panel demonstrated remarkable performance in terms of uniformity and capture efficiency, which are two key factors influencing the GBTS costs. The uniformity reached an impressive value of 98.03%, while the capture efficiency peaked at 74.84%. Additionally, the SNP detection rate between two independent GBTS experiments was found to be 99.34%. AgriPlex Genomics has used 1,039 SNP fresh tomato chips to analyze 2,726 samples and identified 696 markers that display polymorphism, with a mean allele frequency (MAF) of 0.19 ± 0.166 across the genome. Here, the average genome-wide MAF is relatively low at 0.18 ± 0.18, which results from the limited number of tomato samples used. The GenoBaits Tomato 10K panel contains 11,174 background SNPs, known as high-confidence SNPs (hcSNPs), which were sourced from the Illumina SNP database (SNP database1). It is worthwhile to note that over the past few decades, Illumina’s Infinium SNP database has been extensively used for various genotyping and SNP chip development in tomatoes (Rodríguez et al., 2013; Hirakawa et al., 2013; Ruggieri et al., 2014; Tong et al., 2023). Sim et al. (2012b) have used 7,720 Illumina SNPs to genotype 426 tomato accessions, thus resulting in the detection of 6,374 polymorphic SNPs across the entire sample, with 6,022 specific to the fresh market tomatoes. Here, 6,768 polymorphic SNPs are identified using 335 fresh market tomato samples. In terms of chromosome coverage rates, the 7,720 Illumina tomato SNP chip has demonstrated an average genome-wide gap of 0.45 Mbp, 0.67 Mbp, and 0.71 Mbp across three distinct F2 populations of 79, 160, and 183 individuals in size, respectively (Sim et al., 2012b). Moreover, AgriPlex Genomics has used the 1K tomato SNP chip to analyze 2,726 tomato samples and reported an average chromosome gap of 0.2 Mbp. Here, the GenoBaits Tomato 10K panel has shown a significantly lower average chromosome gap of 0.07 Mbp, importantly contributed by the number of SNPs. Overall, the GenoBaits Tomato 10K panel has shown outstanding performance, making it a highly suitable SNP panel for genetic analysis in tomato.

The GenoBaits Tomato 10K panel is effective for genetic analyses in tomatoes, as it produces comparable results in genetic diversity, population structure, and PCA analyses when using both trait-specific markers and the 10K panel. This indicates that the panel’s performance is not affected by trait-specific marker sites. The kinship results from the GenoBaits Tomato 10K panel show inconsistencies when compared to those from the trait-specific panel. These discrepancies arise because the trait-specific markers are limited in number and unevenly distributed across the 12 tomato chromosomes as highlighted by the linkage disequilibrium (LD) decay distance. To elucidate population relatedness, Kristen et al. (2011) have established some range values. Based on these classifications, both the trait-specific markers and the 10K panel have categorized the tomato samples as a half-sibling population. This considerable relatedness among the tomato samples has compromised the PIC values for both the background SNPs and trait-specific markers. To our knowledge, the tomato lines used in this study are derived from a few elite commercial hybrid varieties, meaning that they may have a restricted genetic background. Further research to explore the high-resolution aspect of this panel is recommended using a larger population that includes wild accessions, cherry tomatoes, fresh market tomatoes, and processing tomatoes. There are several factors that influenced our choice of the tomato genotype panel for this study. Firstly, as a private entity, Molbreeding Biotechnology Co., Ltd encounters difficulties in accessing a diverse array of genetic varieties. As a result, the genotype panel we employed was generously supplied by several of our commercial partners. Secondly, the tomato lines used in this research are derived from elite commercial varieties known to harbor various resistance genes. We believe these lines will be instrumental in genotyping trait-specific marker sites associated with resistance. Finally, the background SNPs of the GenoBaits Tomato 10K panel were compiled using various SNP modules, primarily sourced from Illumina’s Infinium SNP database, which is widely recognized in numerous SNP genotyping projects for tomatoes. Therefore, we expect that many of the SNPs in this 10K panel will provide valuable insights when applied to a broad range of genetic backgrounds. In conclusion, the choice of this tomato genotype panel is mainly driven by two important factors such as genetic diversity and the identification of resistance loci.

In this study, trait-specific marker sites associated with disease resistance and other desirable traits, such as abiotic stress tolerance, fruit quality, and yield, are successfully identified. Tomato is facing more than 60 pathogens that negatively impact tomato production worldwide (Jones et al., 2014; El-Sappah et al., 2022; Salem et al., 2023; Zhang et al., 2022; Ghorbani, 2024), and among them, at least 10 have been extensively reported in China (Yan et al., 2018). Interestingly, the GenoBaits Tomato 10K panel could simultaneously diagnose 19 resistance genes associated with 10 tomato diseases using GBTS technology. We recommend validating those resistance marker sites in the future to improve the potential use of this panel in tomato backcross breeding, gene pyramiding, and genomic selection studies.

## Conclusion

This study demonstrates the performance of the GenoBaits Tomato 10K panel, emphasizing both background SNPs and trait-specific markers associated with disease resistance, abiotic stress tolerance, fruit quality, and yield. The panel effectively evaluates genetic diversity and validates 19 disease resistance loci, making it a valuable diagnostic resource for tomato genomics and breeding research. However, the limited background of the tomato sample panel has somewhat diminished the average polymorphism information content. Therefore, we recommend future studies include wild accessions, cherry tomatoes, fresh market varieties, and processing types to identify more informative SNPs and enhance the panel’s applicability. Additionally, we propose the newly developed panel for use in tomato backcross breeding, gene pyramiding, and genomic selection research.

## Data Availability

Publicly available datasets were analyzed in this study. This data can be found here: Database1: https://pmc.ncbi.nlm.nih.gov/articles/PMC3393668/ Database2: https://pmc.ncbi.nlm.nih.gov/articles/PMC3686429/ Database3: https://pmc.ncbi.nlm.nih.gov/articles/PMC3859326/ Database4: https://www.ncbi.nlm.nih.gov/sra/?term=SRP150040.
