# Supplementary figures and images for: An advanced 10K SNP panel for genotyping tomato (Solanum lycopersicum L.) via targeted genome sequencing
Source: Front Plant Sci. 2025 May 21;16:1582241. doi: 10.3389/fpls.2025.1582241 (PMC12133979; doi:10.3389/fpls.2025.1582241)

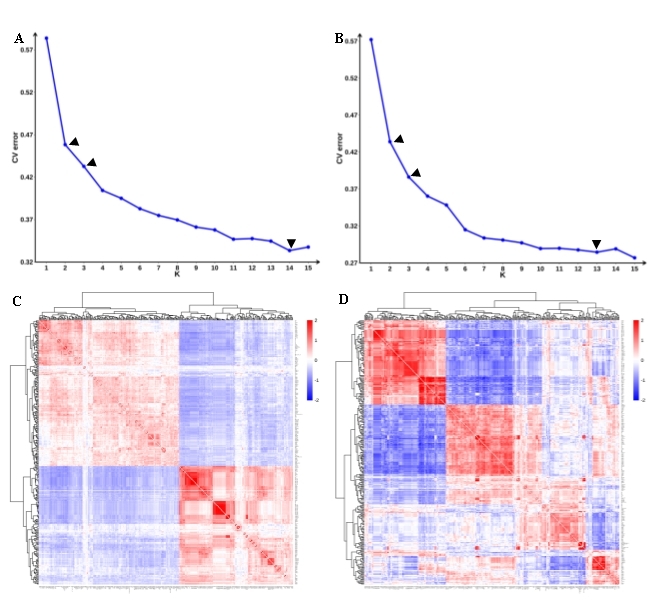

Supplement: Supplementary file 1 [file SupplementaryFile1.jpg]
